# 97. Tetravalent Dengue Vaccine (TAK-003) Development Program: A Bird’s Eye View

**DOI:** 10.1093/ofid/ofab466.097

**Published:** 2021-12-04

**Authors:** Vianney Tricou, Shibadas Biswal, Mengya Liu, Sanjay S Patel, Olaf Zent, Martina Rauscher, Gonzalo Perez, Walid Kandeil, Nicolas Folschweiller

**Affiliations:** 1 Takeda Pharmaceuticals, Zurich, Zurich, Switzerland; 2 Takeda Pharmaceuticals International AG, Zurich, Zurich, Switzerland; 3 Takeda Phrmaceuticals, lexington, Massachusetts; 4 Takeda, Zurich, Zurich, Switzerland

## Abstract

**Background:**

Dengue fever is a mosquito-borne viral disease endemic in 128 countries. An unmet clinical need remains for an effective vaccine that can be used more broadly than the vaccine presently available. A clinical development program has evaluated the long-term safety, immunogenicity, and vaccine efficacy (VE) of TAK-003, a live attenuated tetravalent dengue vaccine with a DENV-2 backbone engineered to elicit immune responses to all 4 dengue serotypes.

**Methods:**

18 clinical trials in 13 countries have involved 28,175 seropositive/seronegative participants aged from 1.5-60 years from endemic/non-endemic regions. In the ongoing pivotal phase III study, 4–16-year-old healthy children (N=20,099) were randomized 2:1 to receive two doses of TAK-003 or placebo, 3 months apart for an evaluation of VE and safety over a multi-year period stratified pre-vaccination dengue serostatus. Active surveillance throughout the trial detected symptomatic dengue. The trial will continue up to 4–4.5 years post 2^nd^ dose, and for another 25 months after a booster dose. Data up to 3 years after the second vaccination are currently available.

**Results:**

Safety and immunogenicity data from Phase I/II studies established the final formulation and dosing schedule. Overall VE in the pivotal phase III study was 80.2% [95% CI: 73.3–85.3] against virologically confirmed dengue (VCD) at 12 months post 2^nd^ dose. At 18 months, VE was 66.2% (95% CI: 49.1–77.5) in dengue-naive and 76.1% (95% CI: 68.5–81.9) in dengue pre-exposed participants, with VE of 90.4% (95% CI: 82.6–94.7) and 85.9% (95% CI: 31.9–97.1) for prevention of hospitalized VCD and dengue hemorrhagic fever, respectively. Cumulative VE against VCD from first dose to 3 years post 2^nd^ dose was 62.0% (95% CI: 56.6–66.7) and 83.6% (95% CI: 76.8–88.4) in prevention of hospitalized VCD. Some decline in VE was observed over time mainly driven by outpatient dengue. Two doses of TAK-003 3 months apart were well-tolerated with no important safety risks identified up to 3 years after completion of the vaccination schedule.

**Conclusion:**

TAK-003 is immunogenic against all 4 dengue serotypes and continues to be efficacious, well-tolerated, and with no evidence of disease enhancement in seronegative population up to 3 years post-vaccination.

**Disclosures:**

**Vianney Tricou, D Phil**, **Takeda Pharmaceuticals International** (Employee) **Shibadas Biswal, MD**, **Takeda Vaccines, Inc** (Employee) **Sanjay S. Patel, PhD**, **Takeda Pharmaceuticals International AG** (Employee) **Olaf Zent, MD**, **Takeda Pharmaceuticals International AG** (Employee) **Martina Rauscher, PhD**, **Takeda Pharmaceuticals International AG** (Employee) **Gonzalo Perez, MD**, **Takeda group companies** (Employee) **Walid Kandeil, MD**, **Takeda Pharmaceuticals International AG** (Employee) **Nicolas Folschweiller, PhD**, **Takeda** (Employee)

